# Screening to Identify an Immune Landscape-Based Prognostic Predictor and Therapeutic Target for Prostate Cancer

**DOI:** 10.3389/fonc.2021.761643

**Published:** 2021-11-05

**Authors:** Yanting Shen, Huan Xu, Manmei Long, Miaomiao Guo, Peizhang Li, Ming Zhan, Zhong Wang

**Affiliations:** ^1^ Department of Urology, Shanghai Ninth People’s Hospital, Shanghai Jiaotong University School of Medicine, Shanghai, China; ^2^ Department of Pathology, Shanghai Ninth People’s Hospital, Shanghai Jiaotong University School of Medicine, Shanghai, China; ^3^ The Core Laboratory in Medical Center of Clinical Research, Department of Endocrinology, Shanghai Ninth People’s Hospital, State Key Laboratory of Medical Genomics, Shanghai Jiao Tong University School of Medicine, Shanghai, China

**Keywords:** prognostic predictor, immune infiltration, progression-free survival (PFS), overall survival (OS), prostate cancer

## Abstract

**Objectives:**

Existing prognostic risk assessment strategies for prostate cancer (PCa) remain unsatisfactory. Similar treatments for patients at the same disease stage can lead to different survival outcomes. Thus, we aimed to explore a novel immune landscape-based prognostic predictor and therapeutic target for PCa patients.

**Methods:**

A total of 490 PCa patients from The Cancer Genome Atlas Project (TCGA) cohort were analyzed to obtain immune landscape-based prognostic features. Then, analyses at different levels were performed to explore the relevant survival mechanisms, prognostic predictors, and therapeutic targets. Finally, experimental verification was performed using a tissue microarray (TMA) from 310 PCa patients. Furthermore, a nomogram was constructed to provide a quantitative approach for predicting the prognosis of patients with PCa.

**Results:**

The immune landscape-based risk score (ILBRS) was obtained. Then, VAV1, which presented a significant positive correlation with Treg infiltration and ILBRS, was screened and identified to be significantly related to the prognosis of PCa. Finally, experimental verification confirmed the prognostic value of VAV1 for PCa prognosis at the protein level.

**Conclusions:**

VAV1 has the potential to be developed as an immune landscape-based PCa prognostic predictor and therapeutic target and will help improve prognosis by enabling the selection of individualized, targeted therapy.

## Introduction

Prostate cancer (PCa) is the most common malignancy of the male reproductive system. Its incidence ranks second only to lung cancer among male malignancies worldwide (1). Although there are some curable therapeutic methods, such as radical prostatectomy (RP), a high recurrence rate still exists due to the biological characteristics of malignant tumors, distant micrometastases, and focal residuals (2). Salvage therapy performed in the early stage of recurrence can reduce the distant metastatic rate, prolong survival, and even cure tumors (3). Therefore, identifying PCa recurrences early greatly reduces mortality and improves patient prognosis.

In clinical practice, serum prostate-specific antigen (PSA), Gleason scores (GS), and pathological TNM (pTNM) staging are commonly used to evaluate recurrence and predict the prognosis of PCa patients. However, they have some limitations. The rising serum PSA level after curable treatment, defined as biochemical recurrence (BCR), is unreliable for predicting PCa patient prognoses because some benign conditions can mimic BCR (4). For many men, BCR does not mean that they are at a high risk of death from PCa (4). GS and pTNM staging are limited by the subjective nature of their assessment, distant micro-metastasis, and variations among patients with the same tumor stage or GS. Recently, immune infiltration has become a rapidly growing field of research to identify special immune cells and their relevant molecules for evaluating the prognosis of various cancers, such as gastric cancer, ovarian cancer, and melanoma (5–9). Some studies have reported PCa by estimating immune cell infiltration patterns (5, 9, 10). However, most of these studies focused on BCR (5, 10), which could limit the prediction power to identify PCa patients with poorer prognoses (4). These studies have provided the motivation and goal for further research exploring credible immune landscape-based prognostic predictors for patients with a high risk of death from PCa.

Progression-free survival (PFS) events were the recommended clinical outcome endpoints of The Cancer Genome Atlas Project (TCGA) database for PCa survival studies (11). It was defined as a new tumor event or death without new tumor events. Therefore, the prognostic predictor constructed using the PFS event would present higher predicted accuracy for identifying patients with an increased risk of death from PCa than those constructed using BCR. In view of this, we chose PFS as a clinical outcome endpoint to establish an immune landscape-based prognostic predictor and therapeutic target for PCa. By identifying patients with a high risk of death from PCa at an early stage, our outcomes would help reduce the mortality of PCa and improve the prognosis of patients.

## Methods

### PCa Gene Expression Dataset

The gene expression data [counts and fragments per kilobase per million (FPKM)] of PCa tissues were downloaded from the TCGA database. FPKM data were transformed into transcripts per million (TPM) values following log_2_ (x + 1) normalization. Count data were used for differentially expressed gene (DEG) analysis. The clinical data for PFS analysis were downloaded from TCGA Pan-Cancer Clinical Data Resource (TCGA-CDR) (11). The PFS analysis integrated TCGA pan-cancer clinical data resources and could drive high-quality survival outcome analytics. Finally, a total of 490 PCa patients from the TCGA cohort were included in the present study. Their clinical features are presented in **Table 1**. Patients with a PFS event were defined as those who had a new tumor event after RP, whether it was a progression of the disease, local recurrence, distant metastasis, new primary tumors at all sites, or died of cancer without a new tumor event, including cases with a new tumor event whose type was N/A (11).

**Table 1:** Clinical features for 490 PCa patients from the TCGA cohort.

Clinical features	Value
Age	Mean +/- standard error (SE): 60.99 +/- 0.309
Gleason score (6/7/8/9/10)	45/244/63/135/3 patients
Distant metastasis	6 patients
Death	4 patients
Death from PCa	2 patients
Patients with PFS event	89 patients
Prior treatment	Not mentioned
Radiation therapy (follow-up)	23 patients
Pharmaceutical therapy (follow-up)	23 patients
Radiation therapy (new tumor event)	24 patients
Pharmaceutical therapy (new tumor event)	22 patients

### Establishment of Immune Landscape-Based Risk Score

First, the immune score was calculated for each PCa tissue in the TCGA cohort using ESTIMATE (12). Then, PCa tissue samples were classified into two groups, the low immune score group and the high immune score group, according to the optimal cutoff value determined by X-tile 3.6.1 software (Yale University, New Haven, CT, USA). DEG analysis between these two groups was performed using the “EdgeR” package (13) using R software 4.0.5, and genes with |log_2_ fold change| > 1 and Benjamini–Hochberg-adjusted *p* < 0.01 were considered immune landscape-based DEGs (IL-DEGs). Subsequently, PFS analyses for these IL-DEGs *via* univariate Cox regression were performed using the Kaplan–Meier function in the R software 4.0.5 survival package. Statistical significance was set at *p* < 0.05. Finally, stepwise Cox regression was used to establish the immune landscape-based risk score (ILBRS) for PFS in patients with PCa. Moreover, a Kaplan–Meier curve was drawn to assess its predictive ability.

### Identification of ILBRS-Relevant Cellular and Molecular Signatures

For the ILBRS-relevant cellular signature, CIBERSORT (14) was used to estimate the proportions of 22 immune cell types in each PCa tissue sample of patients in the TCGA-PRAD cohort. Then, PFS analysis, *t*-test, and Pearson correlation analysis were performed to evaluate the relationship between immune infiltration and ILBRS. For the ILBRS-relevant molecular signature, PCa tissue samples were reclassified into low ILBRS and high ILBRS groups according to the optimal cutoff value of ILBRS determined by X-tile 3.6.1 software (Yale University, New Haven, CT, USA) (15). Then, gene set enrichment analysis (GSEA) was performed between these two groups to identify the significantly enriched immune-relevant KEGG pathways (normal *p* (NP) < 0.01 and false discovery rate (FDR) < 0.05). Finally, single-sample GSEA (ssGSEA) was performed to estimate the enrichment score (ES) of KEGG pathways for each PCa tissue sample. Gene set variation analysis (GSVA), PFS analysis, and Pearson correlation analyses were performed to identify ILBRS-relevant molecular mechanisms and therapeutic targets.

### Immunohistochemical Analysis

Samples of tissue microarray (TMA) were obtained from patients with PCa who underwent RP between January 2008 and December 2018 at the Department of Urology of Shanghai Ninth People’s Hospital, Shanghai Jiaotong University School of Medicine. All patients were informed of the importance of follow-up and were regularly followed up. Overall survival was defined as the time interval between surgery and the last follow-up (December 31, 2019) or death. Clinical information is shown in **Table S1**. All paraffin tissue sections obtained from the TMA were dewaxed and rehydrated. After antigen retrieval and blocking with bovine serum albumin (Sango Biotech, Shanghai, China), the slides were incubated with anti-VAV1 (1:50, Cat. #HPA001864, Sigma-Aldrich, St. Louis, MO, USA) overnight at 4°C. Then, they were incubated with a goat anti-rabbit horseradish peroxidase-conjugated secondary antibody (Cell Signaling Technology, Beverly, MA, USA) for 1 h at 25°C. DAB solution was used for brown color development. Quantification of immunohistochemical (IHC) staining was based on the staining intensity (I score: negative, 0; weak, 1; moderate, 2; and intense, 3) and the percentage of positively stained cells (P score: 0%–5%, a score of 0; 6%–35%, a score of 1; 36%–70%, a score of 2; and >70%, a score of 3). The final score was obtained by using the formula Q score = I score × P score. Samples with Q scores of ≥ 4 were considered highly expressed, while those with Q scores < 4 were considered to have low expression. IHC staining results were independently evaluated by at least two senior pathologists.

### Nomogram Construction and Evaluation

We further used the coefficients of the multivariable Cox regression model to formulate a nomogram using the “rms” package (16) in R software 4.0.5. The 5-year calibration curves were assessed graphically by plotting the observed rates against the nomogram-predicted probabilities. A concordance index (C-index) was calculated using a bootstrap method with 1,000 resamples to determine the discrimination of the nomogram.

### Statistical Analysis

Statistical analyses were performed using R software (version 4.0.5). The *χ^2^*-test was used for risk assessment. Pearson’s correlation analysis was performed to determine the correlation between the two variables. PFS analysis *via* the Kaplan–Meier method was performed using Log Rank (Mantel–Cox) to evaluate long-term PFS and Breslow (Generalized Wilcoxon) to evaluate short-term PFS. Statistical significance was set at *p* < 0.05.

## Results

### Patients With High Immune Scores Had a Poorer PFS, Suggesting That Immune Landscape Affected PCa Prognosis

**Figure 1:**
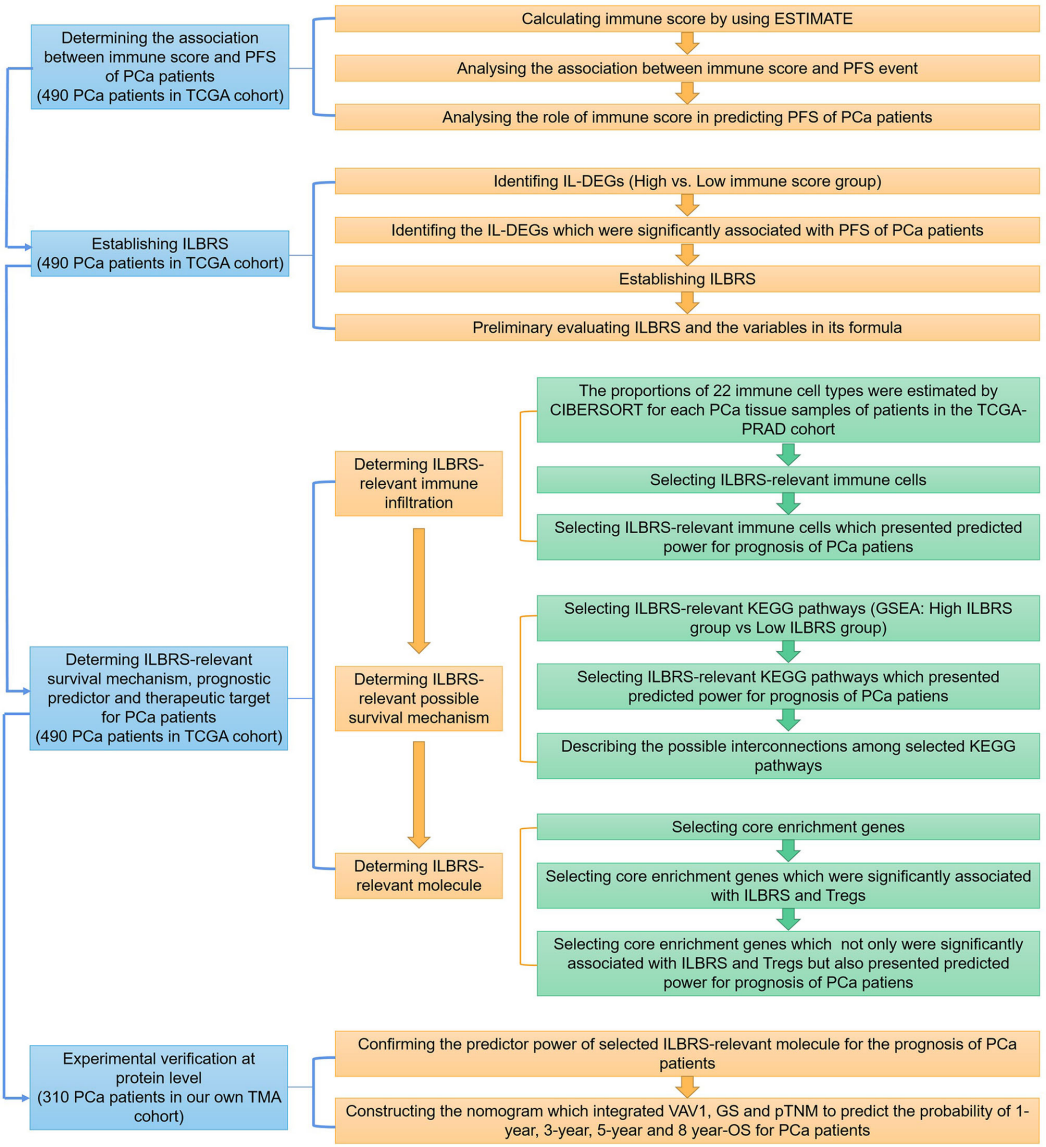
The detailed strategy of discovering the immune landscape-based prognostic predictor and therapeutic target for prostate cancer.

In this study, three major steps were performed to uncover the immune landscape-based prognostic signature for PCa: establishing ILBRS, determining the ILBRS-relevant underlying survival mechanism, and conducting the experimental verification of the ILBRS-relevant key molecule. A detailed strategy is shown in **Figure 1**. A total of 800 PCa data points were included in the present study. Expression data of genes were from the TCGA-PRAD cohort, and those of proteins were obtained from our cohort.

**Figure 2:**
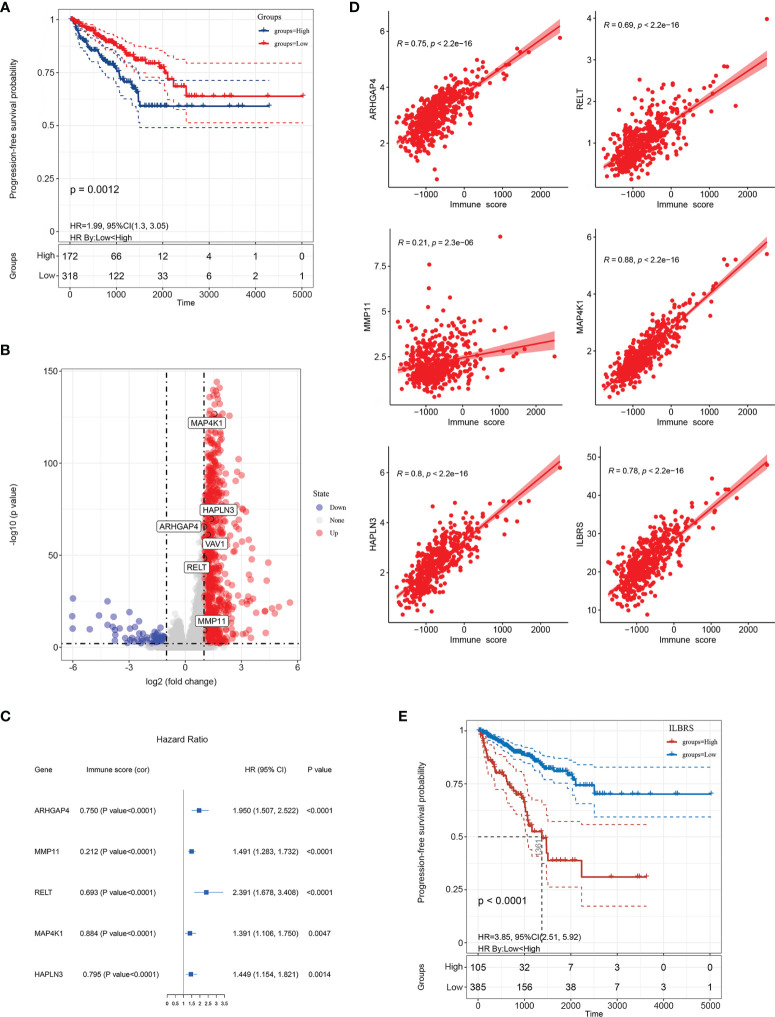
Establishment of immune landscape-based risk score (ILBRS). **(A)** Kaplan–Meier curves for high and low immune score patient groups in TCGA-PRAD data. **(B)** Volcano plot of immune landscape-based DEGs (IL-DEGs). **(C)** Forest plot of the results of univariate Cox regression analyses of IL-DEGs included in the ILBRS formula. The square data markers indicate the estimated hazard ratios (HRs). Error bars represent 95% confidence intervals (CIs). “cor” indicates the coefficient gained through Pearson correlation analysis. **(D)** Pearson correlation analysis of ILBRS and its variables with immune scores. **(E)** Kaplan–Meier curves for high and low ILBRS patient groups in TCGA-PRAD data.

First, the immune score representing the immune landscape was calculated for 490 PCa tissues in the TCGA-PRAD cohort by ESTIMATE. This method used gene expression signatures to infer the fraction of immune cells and determined the immune score *via* ssGSEA (**Table S2**) (11). Then, X-tile software was used to choose the best cutoff value to divide these 490 PCa tissues into high and low immune score groups. As expected, the results of risk assessment and PFS analysis *via* the Kaplan–Meier method showed that patients with high immune scores had a higher risk for PFS events (*χ^2^* = 10.826, *p* = 0.001, OR = 2.190, 95% CI = (1.364–3.518)) and poorer short-term and long-term PFS than patients with low immune scores (Log Rank [Mantel–Cox]: *χ^2^* = 10.461, *p* = 0.001; Breslow (Generalized Wilcoxon): *χ^2^* = 12.199, *p* < 0.0001; **Figure 2A**), indicating that immune score was a risk factor for PFS events and significantly affected the prognosis of PCa patients.

### ILBRS, the Prognostic Signature for PFS of PCa Patients, Was Obtained

In order to establish the ILBRS to describe the immune landscape-based prognostic signature for PCa, four major steps were performed: identification of IL-DEGs, PFS analysis of IL-DEGs *via* the Kaplan–Meier method and univariate Cox regression, establishment of ILBRS *via* stepwise Cox regression multivariate analysis, and preliminary evaluation of ILBRS and the variables in its formula.

Gene expression differences were compared between the groups with high immune and low immune scores, and 1,907 IL-DEGs were identified. Of these, 934 were coding genes (**Figure 2B**). After filtering low-abundance genes, the average expression level was lower than 0.01, and 415 IL-DEGs were selected for subsequent PFS analyses. The results of PFS analysis *via* univariate Cox regression showed that 137 IL-DEGs played a significant role in predicting PFS in PCa patients (**Table S3**). Among them, 135 IL-DEGs were chosen for further stepwise Cox regression multivariate analysis, which was used to screen the optimal combination and establish ILBRS (two IL-DEGs (*ALB* and *LCN2*) were excluded because of the opposite results of their DEGs analysis and PFS analysis). The results are presented in **Table 2**. ILBRS was established using five IL-DEGs including RELT TNF receptor (*RELT*), matrix metallopeptidase 11 (*MMP11*), Rho GTPase activating protein 4 (*ARHGAP4*), mitogen-activated protein kinase 1 (*MAP4K1*), and hyaluronan and proteoglycan link protein 3 (*HAPLN3*) (Omnibus test: *p* < 0.0001). The formula for calculating ILBRS for each patient was as follows: ILBRS = (2.816 * expression level of *RELT*) + (1.318 * expression level of *MMP11*) + (4.774 * expression level of *ARHGAP4*) + (0.393 * expression level of *MAP4K1*) + (0.613 * expression level of *HAPLN3*). Preliminary evaluation of ILBRS and IL-DEGs in its formula was performed. As shown in **Figure 2C**, the expression of five IL-DEGs was upregulated significantly, increasing the immune score. Except for *MMP11*, all IL-DEGs and ILBRS exhibited a strong positive correlation with the immune score (**Figure 2D**). Then, the optimal cutoff value (26.9) chosen by the x-tile software was used to regroup the 490 patients in the TCGA-PRAD cohort into high and low ILBRS groups. As shown in **Figure 2E**, the patients with high ILBRS had poorer short-term and long-term PFS than those with low ILBRS (Log Rank [Mantel–Cox]: *χ^2^* = 44.085, *p* < 0.0001; Breslow (Generalized Wilcoxon): *χ^2^* = 37.901, *p* < 0.0001). These results suggest that ILBRS and the variables in its formula are robust immune landscape-based prognostic signatures for PFS in PCa patients.

**Table 2:** Immune landscape-based DEGs (IL-DEGs) included in the formula of the immune landscape-based risk score (ILBRS).

IL-DEGs	B	Standard deviation (SD)	*p*-value	Coefficient	95% Confidence interval (CI)	
					Upper limits	Lower limits
RELT	1.035	0.340	0.002	2.816	1.445	5.489
MMP11	0.276	0.090	0.002	1.318	1.105	1.573
ARHGAP4	1.563	0.335	0.000	4.774	2.475	9.209
MAP4K1	-0.934	0.283	0.001	0.393	0.226	0.685
HAPLN3	-0.490	0.220	0.026	0.613	0.398	0.944

### *VAV1*, ILBRS-Relevant Predictor and Therapy Target for PFS of PCa Patients, Was Identified

**Figure 3:**
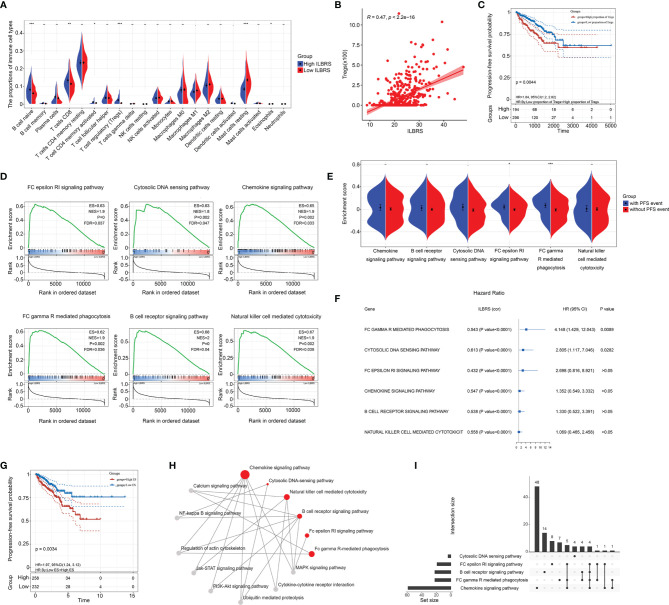
ILBRS-relevant immune cell infiltration and immune-relevant KEGG pathways. **(A)** The proportions of 22 immune cell types in high and low ILBRS patient groups in TCGA-PRAD data. **(B)** Pearson correlation analysis of ILBRS and Tregs infiltration. **(C)** Kaplan–Meier curves for the PCa tissues from patients with high and low Tregs infiltration in TCGA-PRAD data. **(D)** KEGG pathway analyses show a notable pathway of the gene signature. **(E)** PFS event risk assessment for the ES of the immune-relevant KEGG pathways. **(F)** Forest plot of the results of univariate Cox regression analyses of the ES of the immune-relevant KEGG pathways. The square data markers indicate estimated hazard ratios (HR). The error bars represent 95% CIs. “cor” shows the coefficient gained through Pearson correlation analysis. **(G)** Kaplan–Meier curves for the PCa tissues from patients with high and low ES of FC gamma R-mediated phagocytosis in TCGA-PRAD data. **(H)** The immune-relevant KEGG pathway network. The red dots represent the six pathways. The gray dots are the intermediate pathways between two of the immune-relevant KEGG pathways. **(I)** Venn plot presenting overlapped genes among the immune-relevant KEGG pathways. *p < 0.05; **p < 0.01; ***p < 0.0001.

The underlying ILBRS-relevant survival mechanisms and therapeutic targets were further explored at different levels. For analyses at the cellular level, the proportions of 22 immune cell types in each PCa tissue sample of patients in the TCGA-PRAD cohort were estimated by CIBERSORT. The results showed that 21 immune cell types were found in PCa tissues (**Table S4**). Naive B-cells, CD8^+^ T cells, activated CD4^+^ T cells (memory), Tregs, eosinophils, and mast cells significantly differed in their proportions in PCa tissues from patients with high ILBRS and low ILBRS. Infiltrations of naive B cells (*p* < 0.0001), CD8^+^ T cells (*p* = 0.002), activated CD4^+^ T cells (memory) (*p* = 0.012), and Tregs (*p* < 0.0001) in patients with high ILBRS were much higher than those in patients with low ILBRS, while infiltration of eosinophils (*p* = 0.037) and mast cells (resting) (*p* < 0.0001) in patients with high ILBRS were less than those in patients with low ILBRS (**Figure 3A**). B-cell-naive, CD8^+^ T-cells, activated CD4^+^ T-cells (memory), Tregs, eosinophils, and mast cells were also significantly correlated with ILBRS based on the results of Pearson correlation analyses (**Table S5**). Among them, naive B cells, CD8^+^ T-cells, activated CD4^+^ T-cells (memory), and eosinophils were shown to be significantly weakly correlated with ILBRS; Tregs (**Figure 3B**) and mast cells (resting) presented significant moderate correlations with ILBRS. Furthermore, the results of univariate Cox regression and Kaplan–Meier analysis showed that only Tregs were well predicted for PFS of PCa patients (**Table S5**); patients with a high proportion of Tregs had poorer short-term and long-term PFS than patients with a low proportion of Tregs (Log Rank [Mantel–Cox]: *χ^2^* = 8.092, *p* = 0.004; Breslow (Generalized Wilcoxon): *χ^2^* = 12.079, *p* = 0.001; **Figure 3C**). Above all, we speculated that Treg infiltration could be a key event in the ILBRS-relevant survival mechanism of PCa.

For analyses at the molecular level, two steps were performed. First, GSEA analysis was used to explore the ILBRS-relevant molecular mechanisms. The results showed that six immune-relevant KEGG pathways, including the B cell receptor signaling pathway, the chemokine signaling pathway, the FC epsilon RI signaling pathway, FC gamma R-mediated phagocytosis, natural killer cell-mediated cytotoxicity, and the cytosolic DNA sensing pathway, were significantly enriched between the high ILBRS group and the low ILBRS group (**Table S6**). All of them were upregulated in the tissues of PCa patients with high ILBRS (**Figure 3D**). The KEGG natural killer cell-mediated cytotoxicity was not included in the subsequent analyses because there was no difference in activated NK cell infiltration between the high and low ILBRS groups (**Figure 3A**). Analyses were performed based on ssGSEA, GSVA, and PFS to evaluate the association between the five immune-relevant KEGG pathways and PFS events. As shown in **Figures 3E–G**, the ES of FC gamma R-mediated phagocytosis was significantly increased not only in patients with PFS events but also in patients with poorer prognoses (Kaplan–Meier method: Log Rank (Mantel–Cox) *χ^2^* = 8.563, *p* = 0.003; Breslow (Generalized Wilcoxon): *χ^2^* = 8.275, *p* = 0.004). Although the five immune-relevant KEGG pathways were significantly associated with ILBRS, FC gamma R-mediated phagocytosis might contribute more to PCa survival.

**Figure 4:**
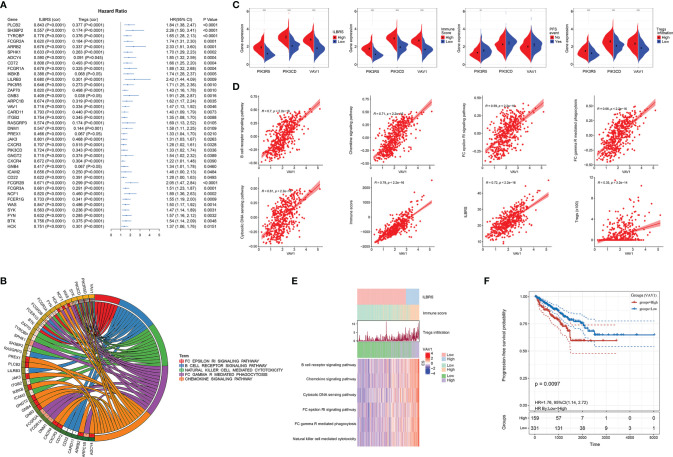
Identification of *VAV1*, an ILBRS-relevant predictor and therapy target for PFS of PCa patients. **(A)** Forest plot of the results of univariate Cox regression analyses of the core enrichment genes of the immune-relevant KEGG pathways. The square data markers indicate estimated hazard ratios (HR). The error bars represented 95% CIs. “cor” indicates the coefficient gained through Pearson correlation analysis. **(B)** The core enrichment genes are associated with PFS of PCa patients in immune-relevant KEGG pathways. **(C)** Different expressions of *VAV1*, *PIK3CD*, and *PIK3R5* between PCa patients with high and low ILBRS, Tregs infiltration, and immune score, and between PCa patients with and without PFS event. **(D)** Pearson correlation analyses of *VAV1* with Tregs infiltration, ILBRS, immune score, and the immune-relevant KEGG pathways. **(E)** Heat map of the immune-relevant KEGG pathways. **(F)** Kaplan–Meier curves for the PCa tissues from patients with high and low expression of *VAV1* in TCGA-PRAD data. *p < 0.05; **p < 0.01; ***p < 0.0001.

Furthermore, KEGG and Venn plots were drawn to describe the possible interconnections among these five immune-relevant KEGG pathways. As shown in **Figures 3H, I**, not all of them were directly connected. Overlapping enrichment genes were common, which were proposed as the link among these five immune-relevant KEGG pathways and had the potential to be developed into ILBRS-relevant prognostic predictors and therapeutic targets for PCa patients. Therefore, a series of analyses focusing on overlapping core enrichment genes were performed. Based on the results of GSEA, 97 core enrichment genes from five immune-relevant KEGG pathways were selected for subsequent analyses (**Table S7**). Univariate Cox regression analysis revealed that 33 core enrichment genes, none of which belonged to the cytosolic DNA sensing pathway, were found to be significantly associated with PFS in PCa patients (**Table S8** and **Figure 4A**). Except for the cytosolic DNA sensing pathway, three core enrichment genes, including *VAV1*, *PIK3R5*, and *PIK3CD*, overlapped among four immune-relevant KEGG pathways (**Figure 4B**). Among them, *VAV1* was identified as the key molecule involved in the ILBRS-relevant survival mechanism due to its significant association with an immune score, ILBRS, Tregs, PFS event, and five immune-relevant KEGG pathways (**Figures 4C–E**). In addition, the results of Kaplan–Meier analysis showed that patients with high *VAV1* expression had poorer short-term and long-term PFS than those with low expression of *VAV1* (log-rank (Mantel–Cox): *χ^2^* = 6.685, *p* = 0.001; Breslow (Generalized Wilcoxon): *χ^2^* = 6.67, *p* = 0.01; **Figure 4F**). Taken together, due to the strong positive correlation with ILBRS, we proposed that *VAV1* could be used instead of ILBRS. Thus, we demonstrated an economical, convenient, and suitable prognostic predictor and therapy target for PCa patients.

### Experimental Verification of VAV1 in PCa TMA

**Figure 5:**
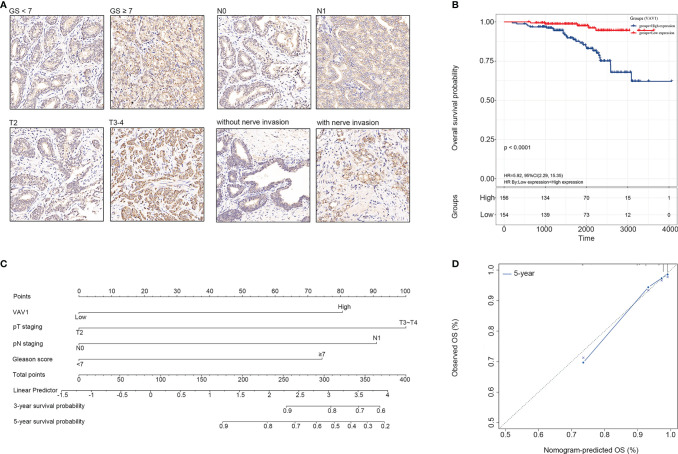
Experimental verification of VAV1 in PCa tissue microarray (TMA). **(A)** The protein expression and localization of VAV1 in PCa TMA. **(B)** Kaplan–Meier curves for the PCa tissues from patients with high and low expression of VAV1 in TMA data. **(C)** Nomogram integrating VAV1, GS, and pTNM to predict the probability of 3- and 5-year OS for PCa patients in TMA data. **(D)** The calibration curve shows that the nomogram model has a better predictive effect on the 5-year OS of PCa patients in TMA data.

*VAV1* is a member of the VAV family of genes. Its coded protein plays an important role in T-cell and B-cell development and activation. Therefore, further experimental verification at the protein level for VAV1 was performed using TMA, including 310 PCa tissue samples. The results showed that a higher expression of VAV1 was significantly associated with GS ≥ 7 (*χ^2^* = 10.419, *p* = 0.001, OR = 2.315, 95% CI = (1.382–3.887)), pT3–pT4 (*χ^2^* = 6.281, *p* = 0.012, OR = 1.996, 95% CI = (1.157–3.444)), lymph node invasion (N1) (*χ^2^* = 8.536, *p* = 0.003, OR = 11.607, 95% CI = (1.480–91.038)), and nerve invasion (*χ^2^* = 13.929, *p* < 0.0001, OR = 2.446, 95% CI = (1.522–3.932)) (**Figure 5A**), indicating that VAV1 expression might affect cell invasiveness. Furthermore, Kaplan–Meier analysis was performed, and the results showed that patients with high VAV1 expression had poorer short-term and long-term overall survival (OS) than those with low VAV1 expression (log rank (Mantel–Cox): *χ^2^* = 17.328, *p* < 0.0001; Breslow (Generalized Wilcoxon): *χ^2^* = 13.227, *p* < 0.0001; **Figure 5B**). Together, these results verified the stability and reliability of VAV1 for predicting the prognosis of PCa patients, which further suggested that it may be developed as a potential therapeutic target for PCa patients with poor prognoses.

In addition, based on multivariate Cox analysis, the 310 samples were also used to construct a nomogram that integrated VAV1, GS, and pTNM to predict the probability of 3- and 5-year OS for PCa patients (**Figure 5C**). The C-index of the predicted model was 0.829. The predictive power of the nomogram model was evaluated and quantified by measuring the degree of fit between the C-index and baseline time predicted by the nomogram in the standard curve. As shown in the calibration curve shown in **Figure 5D**, the nomogram model presented well the predicting value of the 5-year OS of PCa patients.

## Discussion

In recent years, the incidence of PCa has increased. Although there are some curative therapeutic methods, the recurrence rate remains high. However, salvage at an early stage of recurrence can improve PCa prognoses. Therefore, the prediction of recurrence has attracted increasing attention. There are some prediction methods in clinical practice, such as the serum PSA test, GS, and pTNM staging, but limitations exist. After curative treatment, BCR is diagnosed when the serum PSA level rises. However, this does not mean that patients with elevated PSA are at a high risk of death from PCa in the longer term because BCR can be mimicked by some benign conditions (4).

In contrast, GS and pTNM staging are deemed more credible for prognostic risk assessment. However, they depend on pathological examinations, are subjected to subjective judgment, and cannot identify distant micro-metastases. In addition, given the heterogeneous nature of PCa, patients with the same GS and pTNM staging may have different prognoses after receiving the same treatment (17, 18). Thus, a satisfactory prognostic predictor beyond the current risk assessment system is desired to accurately identify patients likely to have poor prognoses, followed by better guide management after curative therapy such as RP (19–21). Recently, some studies indicated that the role of immune cell infiltration and their relevant molecules in evaluating the prognosis of PCa could not be ignored (5, 9, 10). However, the study by Rui lacked experimental validation at the protein level and dismissed patients with recurrences caused by focal residual (9); the studies by Shao (5) and Liu (10) focused on BCR, by which the constructed predictor models would have limited predictive power to identify PCa patients with poorer prognoses (4). Therefore, in the present study, we used well-established TCGA-PRAD cohort data to delineate the immune landscape-based prognostic signature for PCa patients and explore its relevant underlying survival mechanism, predictors, and therapeutic targets through analyses at the cellular and molecular levels. Furthermore, experimental verification was performed to prove our outcome’s stability and reliability at the protein level using TMA data from 310 PCa patients.

Immune scores were calculated for each sample in the TCGA-PRAD cohort, based on which ILBRS was established as the prognostic signature for PFS in PCa patients. The formula contained five genes, *RELT*, *MMP11*, *ARHGAP4*, *MAP4K1*, and *HAPLN3*. Except for *MMP11*, all of them presented a strong positive correlation with the immune score. To our knowledge, only MMP11 and HAPLN3 have been reported as possible diagnostic biomarkers or prognostic predictors for PCa (22–25). There is no research on the roles of ARHGAP4 and HAOPLN3 in PCa, but the existing evidence indicates that their functions in tumor recurrence and metastases should not be ignored. ARHGAP4 has been reported to play an important role in regulating cell migration and invasion in pancreatic cancer (26). MAP4K1 could inhibit T cell function and has been proposed as a promising target for cancer immunotherapy (27, 28). RELT is a member of the TNFR superfamily and is primarily expressed in immune cells and lymphoid tissues. Its immunological function is not well defined, and no relevant study describes its association with malignancy. However, Choi et al. proposed that RELT could act as a negative regulator that controls the early phase of T-cell activation, probably by promoting T-cell apoptosis (29). Therefore, we speculated that RELT might play a role in tumor immunosuppression in PCa. Considered together, ILBSR reflects the immune features of cellular migration, invasion, and tumor immunosuppression.

Cellular and molecular analyses were performed to explore the ILBSR-relevant underlying survival mechanism, prognostic predictor, and therapeutic target for PCa patients. Tregs are immunosuppressive cells that play an important role in tumor immune escape (30). As expected, we noticed that high infiltration was significantly associated with poor prognosis in PCa patients, consistent with Liu et al. (10). In addition, five immune-relevant KEGG pathways and their common core enrichment gene *VAV1* were identified. *VAV1* is a member of the VAV family of genes. Previous studies have shown that VAV1 could promote T cell transformation into Tregs, while Tregs could also indirectly induce macrophage VAV1 which enhances the efferocytosis of macrophages, leading to tumor immune escape (31). Our study found that *VAV1* was a key link connecting Tregs and five immune-relevant KEGG pathways and revealed the features of tumor invasion and immunosuppression. *VAV1* was positively correlated with immune scores, ILBRS, Treg infiltration, and five immune-relevant KEGG pathways, and both it and its coded protein presented significant predictive power for the prognosis of PCa patients. Moreover, VAV1 was significantly associated with GS, pathological T staging, lymph node invasion (pathological N staging), and nerve invasion at the protein level, indicating its effect on tumor cell invasiveness. Taken together, we propose that by combining with Tregs, VAV1 might play an important immunosuppressive role in ILBRS-related survival mechanisms and could be more economical, convenient, and suitable as a prognostic predictor and therapy target for PCa patients.

Finally, to provide clinicians with a quantitative approach for predicting PCa patients’ prognosis, a nomogram that integrated VAV1, GS, pathological T staging, and pathological N staging was constructed. The nomogram was more accurate for predicting short-term and long-term survival in PCa patients than individual prognostic factors.

Although VAV1 has been reported as a predictor for the prognosis of some malignancies (32), its role in PCa survival remains unclear. To our knowledge, our study is the first to report the feasibility and accuracy of VAV1 for determining PCa prognosis. Moreover, given the immune landscape, we propose that VAV1 is the key molecule involved in ILBRS-relevant survival mechanisms, indicating its potential as an immune therapeutic target for PCa patients with poor prognoses. However, this study has several limitations. First, OS is an important clinical outcome endpoint for survival studies, with the advantage that there is minimal ambiguity in defining an OS event. At the same time, it is not recommended for PCa survival studies using the TCGA cohort, where there are only 10 OS events out of 500 cases (11). Therefore, in this study using the TCGA database, PFS was chosen as a substitute for OS to establish ILBRS and identify its relevant key molecule. Although the results of experimental verification using our TMA cohort confirmed the predictive power of VAV1 at the protein level for OS of PCa patients, unpredictable biases may still exist. Second, the biological mechanisms of VAV1 and Tregs involved in the survival mechanism of PCa remain elusive. Further in-depth investigations into their functions should be performed in the future.

In conclusion, VAV1 was identified as a key molecule involved in the underlying immune-relevant survival mechanism in this study. This finding indicates that VAV1 could be an immune landscape-based prognostic predictor and therapeutic target for PCa patients in the future.

## Data Availability Statement

The original contributions presented in the study are included in the article/**Supplementary Material**. Further inquiries can be directed to the corresponding authors.

## Ethics Statement

The studies involving human participants were reviewed and approved by the Ethics Committee of Shanghai Ninth People’s Hospital. The patients/participants provided their written informed consent to participate in this study.

## Author Contributions

ZW and MZ had full access to all of the data in the study and take responsibility for the integrity of the data, the accuracy of the data analysis, and the critical revision of the manuscript for important intellectual content. YS and HX took responsibility for the concept, design, data analysis, and paper written. ML took responsibility for the sample collection, TMA preparation, and pathological diagnosis. ML, MG, and PL took responsibility for the immunohistochemistry and evaluation of immunostaining. All authors contributed to the article and approved the submitted version.

## Funding

This work was supported by the National Science Foundation of China (Nos. 81970656 and 62101319).

## Conflict of Interest

The authors declare that the research was conducted in the absence of any commercial or financial relationships that could be construed as a potential conflict of interest.

The reviewer BD declared a shared affiliation, with no collaboration, with the authors to the handling editor at the time of review.

## Publisher’s Note

All claims expressed in this article are solely those of the authors and do not necessarily represent those of their affiliated organizations, or those of the publisher, the editors and the reviewers. Any product that may be evaluated in this article, or claim that may be made by its manufacturer, is not guaranteed or endorsed by the publisher.

## Abbreviations

ILBRS, immune landscape based risk score; IL-DEGs, immune landscape based DEGs.
