# Supplementary figures and images for: Screening to Identify an Immune Landscape-Based Prognostic Predictor and Therapeutic Target for Prostate Cancer
Source: Front Oncol. 2021 Nov 5;11:761643. doi: 10.3389/fonc.2021.761643 (PMC8602809; doi:10.3389/fonc.2021.761643)

## Slide 1
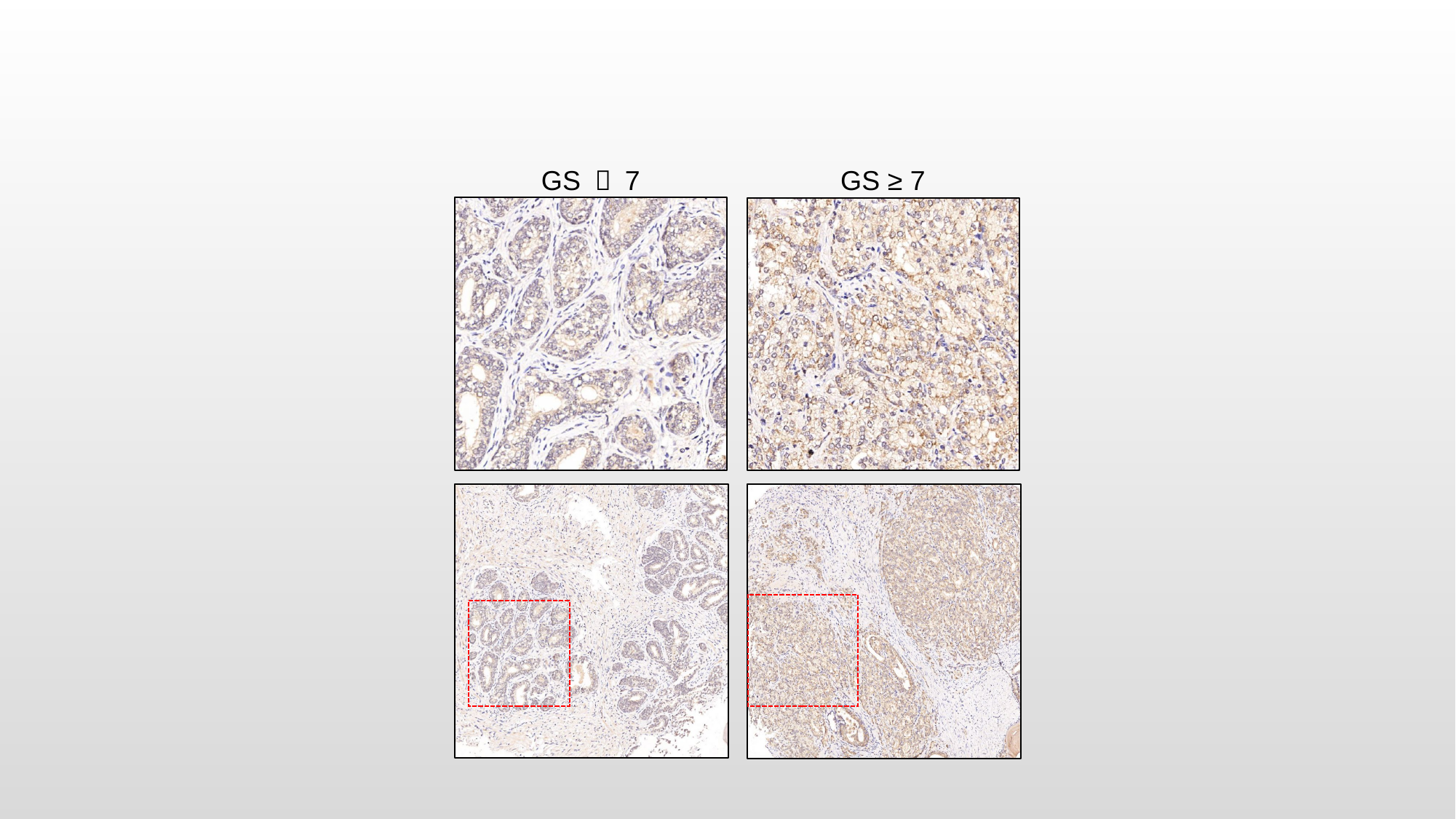

GS ＜ 7
GS ≥ 7

## Slide 2
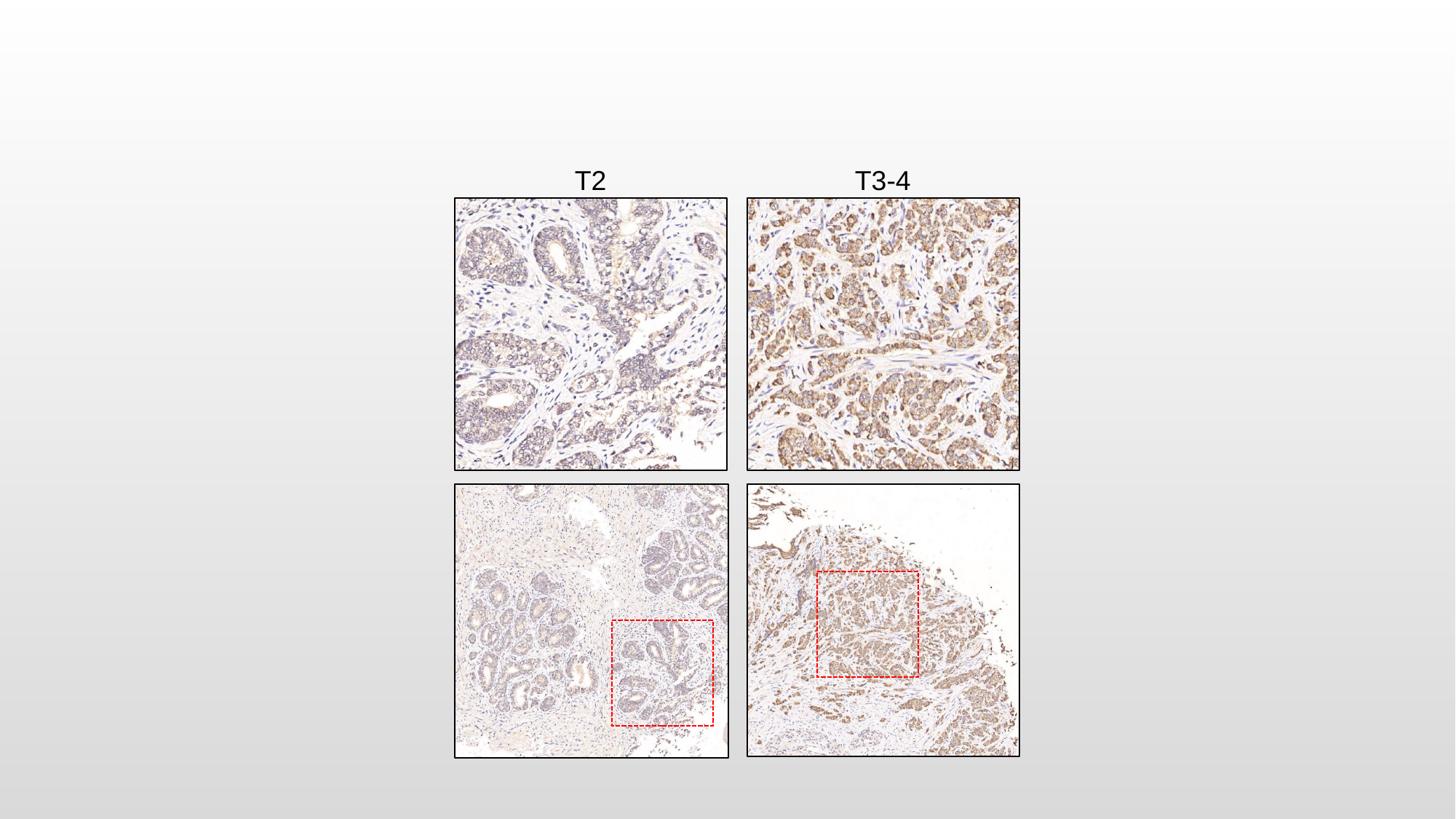

T2
T3-4

## Slide 3
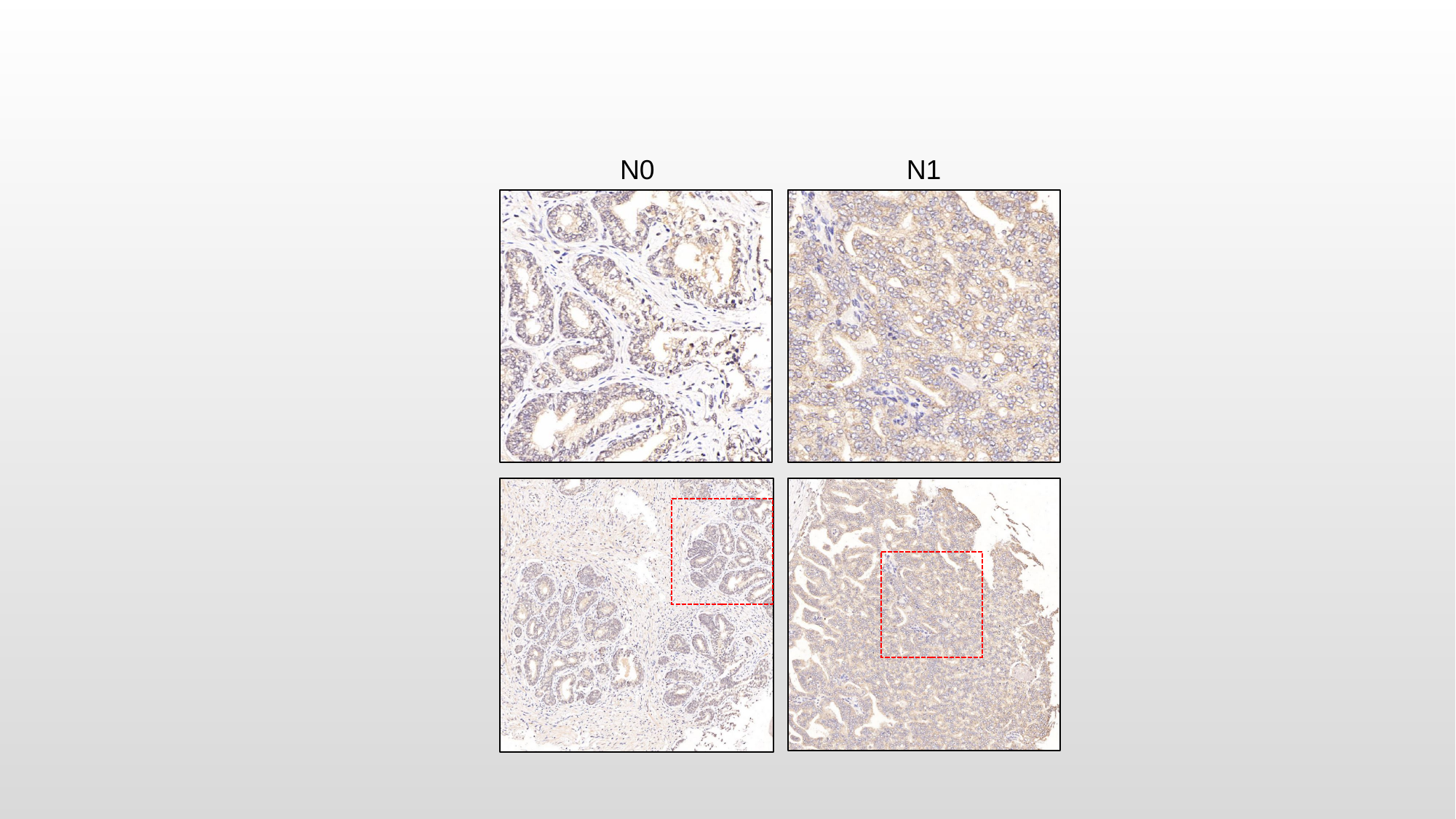

N0
N1

## Slide 4
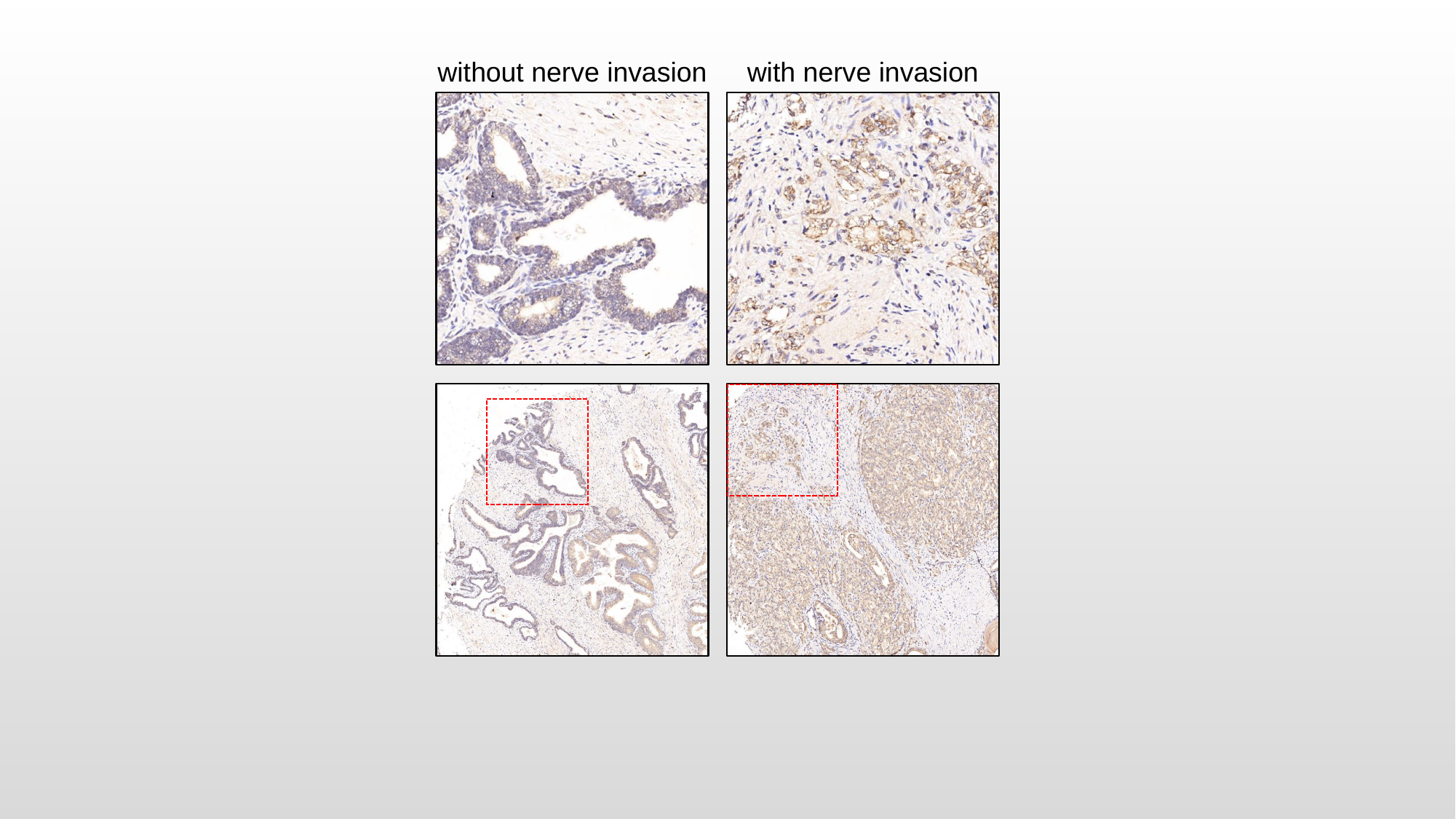

without nerve invasion
with nerve invasion

Supplement: Supplementary file 3 [file DataSheet_3.zip › Data Sheet 3/data2/Figure 5/HIC/Figure5A_VAV1 and PCa.pptx]
